# A Rare Case of Transitional Cell Carcinoma in an Adult Male With Neurofibromatosis Type 1

**DOI:** 10.7759/cureus.18456

**Published:** 2021-10-03

**Authors:** Kunal Aggarwal, Kerry Fine, David Wong

**Affiliations:** 1 Medical Education, Saint George's University School of Medicine, True Blue, GRD; 2 General Surgery, Arrowhead Regional Medical Center, Colton, USA; 3 Surgery, Arrowhead Regional Medical Center, Colton, USA

**Keywords:** neurofibromatosis type 1, transitional cell carcinoma, von recklinghausen disease, bladder, hematuria

## Abstract

Neurofibromatosis type 1 (NF1) is a multisystem genetic disorder characterized by café-au-lait macules on the skin, Lisch nodules of the iris, and predisposition to a wide array of tumors. These include neurofibromas, pheochromocytomas, and gastrointestinal stromal tumors (GIST). While there is documented evidence to suggest that the NF1 gene may play a role in the pathogenesis of transitional cell carcinoma (TCC) of the bladder, there is a paucity of documented cases of TCC in patients with NF1. Our patient is a 53-year-old male with a known diagnosis of NF1 and prior history of GIST who presented to the emergency department with lower abdominal pain, constipation, hematuria, and oliguria. The patient was found to have marked colonic distention prompting a decompressive cecostomy with subsequent return of bowel function. Cystoscopy was performed at this time for hematuria, which revealed a 9 cm bladder mass. Pathology showed a high-grade TCC of the bladder with nuclear pleomorphism and necrosis. The patient was treated with gemcitabine and cisplatin neoadjuvant chemotherapy, followed by cystoprostatectomy with bilateral pelvic lymphadenectomy and ileal conduit urinary diversion. Our case report is the first documented instance in the United States exhibiting an in vivo association of NF1 with the development of TCC of the bladder, an association previously identified in vitro. We hope our work inspires further investigation into this unique association.

## Introduction

Neurofibromatosis type 1 (NF1) is a multisystem autosomal dominant disorder characterized by café-au-lait macules on the skin, skeletal abnormalities, Lisch nodules of the iris, and growth of various tumors [1]. Patients with NF1 are predisposed to a wide array of benign and malignant tumors, most notably neurofibromas. Neurofibromas are benign cutaneous tumors of peripheral nerves that form on or below the skin. These can occasionally progress to malignant peripheral nerve sheath tumors, with a rate of transformation between 2.4% and 16.5% [2]. Other documented neoplasms include pilocytic astrocytomas, pheochromocytomas, juvenile myelomonocytic leukemia, and gastrointestinal stromal tumors (GISTs). The tumors found in these patients are most often due to an inherited defect in the NF1 tumor suppressor gene, which results in the activation of the rat sarcoma virus (RAS) proto-oncogene pathway [3].

There has been extensive research on the role of the NF1 gene in the pathogenesis of many other cancers. There is evidence that suggests somatic NF1 mutations may play a role in the cancers of patients that do not have neurofibromatosis type 1 [4]. One such cancer is transitional cell carcinoma (TCC) of the bladder. A 1999 in vitro study by Aaltonen et al. showed that NF1 mRNA and protein levels were decreased in high-grade TCC of the bladder, ultimately suggesting variable expression of the gene [5].

Literature review for the association of NF1 with TCC of the bladder has only yielded this study from 1999 describing in vitro and in vivo pathogenesis of the NF1 gene and TCC of the bladder [5]. Over 20-years later, we present a unique case of a 53-year-old NF1 patient who was diagnosed with high-grade TCC of the bladder during his hospital course at our institution. Our hospital is a high-volume county teaching hospital in the southern California region.

## Case presentation

Our patient was a 53-year-old male who presented to an outside emergency department with a two-week history of gross hematuria and progressive oliguria. His past medical history included untreated and uncontrolled hypertension, NF1, and a gastrointestinal stromal tumor (GIST) of the small bowel that was surgically resected five years prior. On presentation to the outside hospital, a Foley catheter (Medline, Mundelein, IL) was placed with appropriate urine output. Bladder irrigation was initiated for hematuria. After a two-day hospital course, he was discharged with a referral to urology for outpatient cystoscopy. One day after his discharge, he developed numerous symptoms prompting a visit to our hospital emergency department.

**Figure 1:**
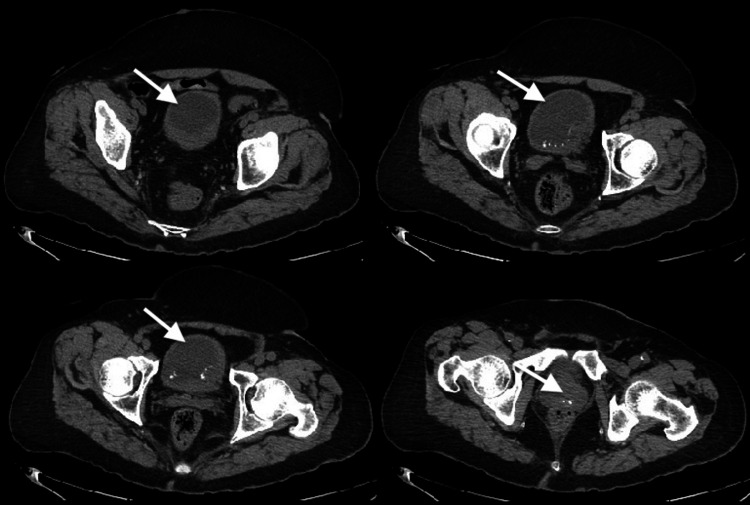
CT pelvis at various levels (white arrows) showing heterogenous hyperdense mass of the bladder suggestive of a bladder tumor with several calcifications.

Upon presentation, the patient complained of dyspnea on exertion, diaphoresis, chills, fatigue, anorexia, lower abdominal pain, and constipation. The patient admitted obstipation for four days. CT scan of the abdomen and pelvis revealed mild right-sided hydronephrosis, diverticulosis in the descending and sigmoid colon, mild prostatomegaly, and a heterogenous hyperdense mass within the bladder (Figure 1). Serial abdominal x-rays indicated a combination of moderate small bowel dilation along with marked colonic distention. The cause of his colonic distension was unclear, although we considered the possibility of colonic mass due to the patient's history. Vitals on admission revealed sinus tachycardia (152 beats per minute) and hypertension (162/123). The patient’s laboratory values indicated sepsis likely secondary to urologic cause with leukocytosis of 18.8 cells/µl, and lactate of 3 mmol/l. Urinalysis showed 3+ protein, 20-50 WBC, and 3+ occult blood with packed RBC’s.

**Figure 2:**
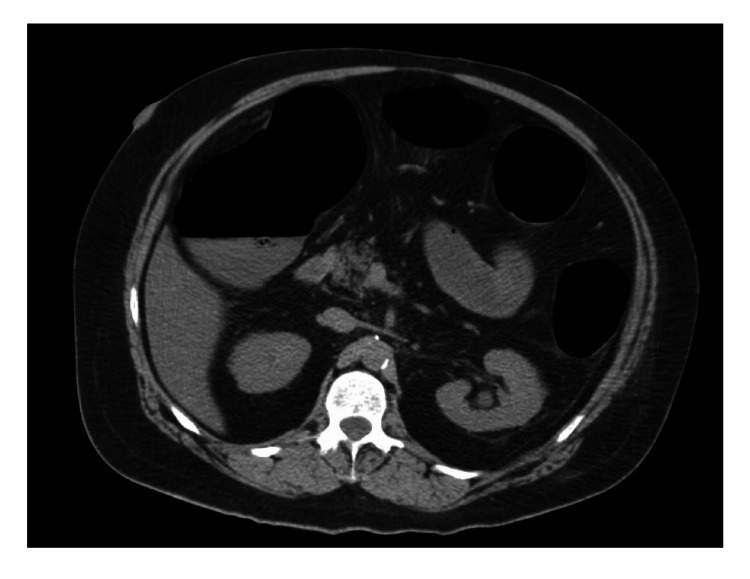
CT abdomen prior to cecostomy showing significantly distended colon suggestive of large bowel obstruction.

**Figure 3:**
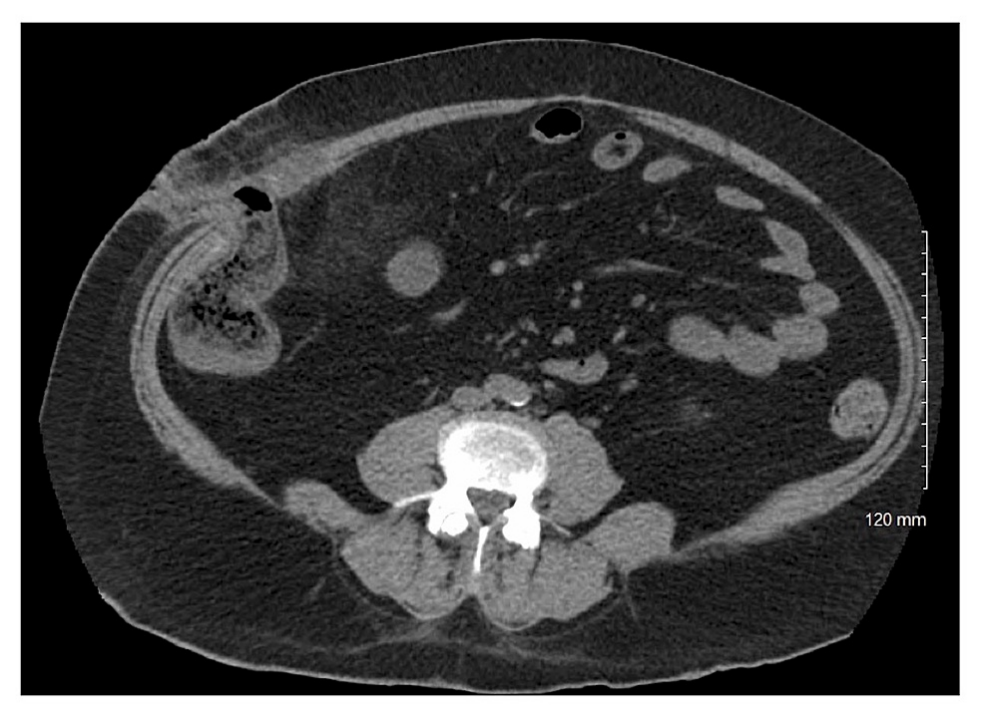
CT abdomen post-cecostomy showing improvement in colonic distention (120 mm ruler for scale).

On the fourth day of the patient’s hospital course, there was no improvement in bowel distension, and he continued to have severe abdominal pain. He underwent an immediate decompressive cecostomy on hospital day five (Figures 2, 3). A distended cecum was noted during the operation with one liter of watery stool released on decompression. Due to the severe distension, surgery was complicated by spillage of bowel contents into the abdominal cavity. The contamination was isolated to the right lower quadrant which was irrigated and evacuated, and the patient was placed on a five-day course of IV cefoxitin post-operatively. There was a mild improvement in abdominal pain following decompression. Output into the cecostomy bag following decompression was minimal despite the patency of the stoma. Due to persistent constipation and large bowel dilatation, there was continued concern for obstruction due to colonic mass or stricture. On hospital day 10, a colonoscopy was performed and revealed normal colonic mucosa and no evidence of any colonic obstruction. Delay in performing colonoscopy occurred due to unremitting obstipation even following decompressive cecostomy and prioritization for treatment of urosepsis.

On hospital day six, a cystoscopy was performed. An extensive bladder mass larger than 9 cm was found extending into the floor and left ventral wall of the bladder. Due to the extent of the tumor, minimal cystoscopic resection was performed and sent for pathology. The biopsy revealed high-grade urothelial carcinoma with nuclear pleomorphism and necrosis. Additionally, immunohistochemical staining for smoothelin demonstrated focal tumor invasion of lamina propria and muscularis propria. Staging workup was performed including chest CT and nuclear medicine bone scan, which were both negative for metastatic disease.

By hospital day 14, the patient started to have small bowel movements from the cecostomy. Repeat abdominal radiograph, performed on day 18, revealed no significant fecal retention or obstruction. On hospital day 15, the patient underwent his first session of neoadjuvant chemotherapy with gemcitabine and cisplatin. The patient was discharged on hospital day 29 with a functional cecostomy. Since his discharge from our institution, the patient has completed a total of four cycles of chemotherapy. Upon completion, he underwent cystoprostatectomy with bilateral pelvic lymphadenectomy and ileal conduit urinary diversion. Cecostomy takedown was performed at this time as well. The patient has since been doing well and continues to follow up in our general surgery clinic.

## Discussion

Information pertaining to the association between NF1 and TCC of the bladder is scarce. Our literature review produced only a single manuscript that outlined the biochemical mechanism involved in the pathogenesis of TCC from loss of function of the NF1 tumor suppressor gene [5]. In the 1999 study, 29 bladder specimens from patients with confirmed TCC were examined. It is worth noting that none of these patients had neurofibromatosis. Specimens ranged from low to high-grade, and were given ratings from one to three with respect to their grade. In 23 of the specimens, there was decreased expression of the NF1 tumor suppressor gene, with a more prominent decrease in grade 3 specimens.

Despite this evidence of an in vitro relationship between the NF1 gene and TCC of the bladder, there are minimal documented cases of patients with this association. The same study cited only two case reports from Japan with this particular presentation. Therefore, our case report is the first documented instance in the United States of an NF1 patient developing TCC of the bladder.

## Conclusions

Our case report further highlights the variably expressive nature of neurofibromatosis type 1. Our patient had multiple well-documented characteristics of NF1 including numerous neurofibromas, hypertension, and a history of GIST of the small bowel. While there is evidence of an association with the NF1 tumor suppressor gene in the pathogenesis of transitional cell carcinoma of the bladder, there is a paucity of documented transitional cell carcinoma of the bladder in patients with neurofibromatosis type 1. Our report complements the larger discussion of effective management of patients with this genetic condition, and more specifically with the diagnosis of transitional cell carcinoma of the bladder. Furthermore, we hope our work inspires further investigation into the association between the NF1 gene and transitional cell carcinoma of the bladder.
